# Retrospective analysis of adjuvant radiotherapy in oral cavity or oropharyngeal cancer: Feasibility of omitting lower-neck irradiation

**DOI:** 10.1371/journal.pone.0266678

**Published:** 2022-04-11

**Authors:** Sheng-Yow Ho, Wan-Chen Kao, Sheng-Yen Hsiao, Sheng-Fu Chiu, Sung-Wei Lee, Jia-Chun Chen, Li-Tsun Shieh

**Affiliations:** 1 Department of Radiation Oncology, Chi Mei Medical Center, Liouying, Tainan, Taiwan; 2 Department of Radiation Oncology, Chi Mei Medical Center, Tainan, Taiwan; 3 Graduate Institute of Medical Science, Chang Jung Christian University, Tainan, Taiwan; 4 Division of Hematology-Oncology, Department of Internal Medicine, Chi Mei Medical Center, Liouying, Tainan, Taiwan; 5 Department of Oral and Maxillofacial Surgery, Chi Mei Medical Center, Liouying, Tainan, Taiwan; IPATIMUP/i3S, PORTUGAL

## Abstract

**Objectives:**

Adjuvant radiotherapy is the standard of care in locally advanced head and neck cancers. The radiation field is correlated with the surgical field in the adjuvant radiotherapy setting; therefore, tailoring the irradiation field is reasonable.

**Materials and methods:**

We retrospectively analyzed patients with oral cavity and oropharyngeal cancers included in the cancer registry between 2015 and 2019 in the study hospital. Patients who underwent whole-neck irradiation (WNI) were compared with those who underwent lower-neck–sparing (LNS) irradiation.

**Results:**

A total of 167 patients with oral cavity and oropharyngeal cancers were included in the study. Cancer recurrence was recorded in 33% of the patients. The rate of recurrence of oral cavity and oropharyngeal cancer at neck level IV was 8%. The 2-year incidence of level IV recurrence was lower in the WNI group than in the LNS group (2% vs. 10%; *p* = 0.04). The 2-year disease-free survival rates were 75% and 63% in the WNI and LNS groups, respectively (*p* = 0.08).

**Conclusion:**

The rate of level IV recurrence was higher in the LNS group than in the WNI group. Trends of improvement in disease-free survival with lower-neck irradiation suggested that it is premature to consider LNS irradiation as daily practice in patients with oral cavity and oropharyngeal cancer.

## Introduction

Most patients with head and neck cancers present with locally advanced, i.e., stage III or IV, disease [1]. Treatment requires multimodality approaches in the majority of these patients. European Society for Medical Oncology (ESMO) has published practice guideline for head and neck cancers in 2020 [2]. Wide excision combined with neck dissection is the standard of care in patients with operable oral cavity disease [2,3], whereas induction chemotherapy is used as a strategy to increase the rate of successful resection in those with unresectable disease [4]. Adjuvant treatment following surgical treatment is often necessary because of the high risk of disease recurrence. Patients with locally advanced oropharyngeal cancer should undergo concurrent chemoradiotherapy as standard of care while surgery followed by radiotherapy or chemoradiotherapy could be considered as an option [2]. Recurrence rate ranges from 45% to 80% in patients who undergo surgery alone [5–7]. Two randomized studies, EORTC 22931 and RTOG 9501, identified several risk factors based on pathology reports and established the role of adjuvant radiotherapy and adjuvant concurrent chemoradiotherapy (CCRT) [8,9]. The rates of local recurrence and distant metastasis relapse following adjuvant radiotherapy are 30% and 25%, respectively [10]. A follow-up program is necessary due to the high probability of cancer recurrence. Physical examination every 3–6 months and imaging such as fiberoptic nasopharyngoscopy and MRI within 3 and 9 months after the end of treatment are strongly recommended [11].

According to the 2021 National Comprehensive Cancer Network (NCCN) guidelines for head and neck cancers, selective neck dissection is recommended for N0 disease, comprehensive neck dissection for N3, and both comprehensive and selective neck dissection for N1 and N2. The extension of selective neck dissection includes at least levels I–III of oral cavity cancer and levels II–IV of oropharyngeal cancer. Considering the relative consistency and predictable pathways of head and neck cancers spreading into neck lymph nodes, neck lymph node levels II to IV are routinely irradiated by volumetric modulated arc therapy [12]. Irradiating level I or not depends on the tumor’s primary site.

Although radiotherapy improved survival outcomes, side effects increased with times. Late radiation-associated dysphagia would negatively impact patient’s quality of life. A multidisciplinary team, including surgeon, radiation oncologist, medical oncologist, radiologist, maxillo-facial prosthodontists and speech-language pathologist is often needed [13]. Furthermore, many studies aimed to reduce damage caused by radiation. A meta-analysis enrolled nine studies investigating node-negative nasopharyngeal cancer (NPC) and concluded that lower-neck–sparing (LNS) irradiation had the same overall survival and disease-free survival (DFS) as levels II–IV neck irradiation [14]. Xiao et al. also mentioned that omitting lower-neck irradiation did not influence oncology outcomes but improved patient-reported voice outcomes [15].

Given that the radiation field largely correlates with the surgical field in the adjuvant radiotherapy setting, irradiating only the nodal region dissected in selective neck surgery is reasonable. Trials on NPC omitting lower-neck irradiation seem to be successful without sacrificing clinical outcomes. Therefore, this study aimed to investigate the irradiation field of lymphatic drainage in those with head and neck squamous cell carcinoma. The pattern of recurrence was evaluated for when lower-neck irradiation was omitted.

## Materials and methods

### Participants

We retrospectively analyzed patients with oral cavity and oropharyngeal cancer in our hospital’s cancer registry from January 2015 to December 2019. Those who underwent curative intent of surgery and adjuvant radiotherapy were evaluated. The inclusion criteria included patients aged older than 20 years, had a good performance status (Eastern Cooperative Oncology Group [ECOG] ≤ 2), and received curative surgery. Conversely, we excluded those who had double cancer, underwent radiotherapy before the diagnosis of oral cavity or oropharyngeal cancer, or did not complete the adjuvant radiotherapy. Patient data such as age, sex, ECOG, primary tumor sites, stage, radiation dose and field, recurrence date, recurrence pattern, survival status, and last follow-up date were recorded from the database. The follow-up time was from the date of cancer diagnosis to December 2020. HR-CTV (clinical target volume at a high risk) was defined as a residual gross lesion or surgical margin–involved site, IR-CTV (CTV at an intermediate risk) referred to the tumor-involved neck and the anatomical structure near the primary lesion, and LR-CTV (CTV at a low risk) was defined as an uninvolved neck. Radiation dose was then recorded as dose to HR-CTV, IR-CTV, or LR-CTV.

### Treatment

All patients underwent tumor-wide excision and neck dissection. Performing comprehensive or selective dissection was at the surgeon’s discretion. The pathologic report determined the need for adjuvant therapy. Patients with major risk factors such as extranodal extension and a positive margin required adjuvant CCRT. Those with minor risk factors listed in the NCCN guidelines underwent adjuvant radiotherapy only. The irradiation field includes the primary tumor site and the neck lymphatic drainage area. Whole-neck irradiation (WNI) referred to irradiation from neck levels Ib–IVa in oral cavity cancer or levels II–IVa in oropharyngeal cancer. LNS referred to the omission of level IV in the irradiation field, that is, irradiation of levels Ib–III in oral cavity cancer or levels II–III in oropharyngeal cancer. The border of neck levels III and IV is the caudate of the cricoid cartilage. Performing WNI or LNS was at the radiation oncologist’s discretion.

### Outcomes

Patients with and without lower-neck irradiation were compared. The primary outcome between these two cohorts was level IV recurrence. The secondary outcomes were DFS, which covered the time from diagnosis to tumor recurrence on the primary site, regional lymphatic drainage, distant metastasis, or death. The potential confounding factors included age, sex, stage, primary tumor site, chemotherapy and performance status (ECOG). The recurrence pattern was also recorded.

### Statistical analysis

Level IV recurrence and DFS between the two cohorts were assessed by Kaplan–Meier analyses with log-rank tests. Patients were censored at the date of death or last follow-up. DFS was further analyzed using Cox regression models, with adjustment for age, sex, primary site of tumor, initial stage, chemotherapy and performance status. The hazard ratios (HR) are expressed in 95% confidence intervals (95% CI). Furthermore, differences in categorical and continuous variables were evaluated using the chi-square and *t*-tests, respectively. All statistical data were analyzed using SPSS version 24.0 (IBM Corp., Armonk, NY, USA). A two-tailed significance level of 0.05 was used. The medical ethics committee of Chimei Hospital approved the research protocol. Given the retrospective design of this study, informed consent was waived.

## Results

From 2015 to 2019, 194 patients who were newly diagnosed with oral cavity and oropharyngeal cancer were identified in our cancer registry database. Among them, 167 were eligible to participate, of which most of them were males (95% [158] vs. 5% [9]). The mean age was 56 (36–92) years, and the median follow-up was 3.26 years. Table 1 summarizes the demographic and clinical characteristics of these patients. The ECOG performance status score of 0–1 accounted for most of the enrolled patients (96%). Oral cavity cancer (i.e., lip, buccal, gingival, retromolar trigone, tongue, and mouth floor tumors) was more common than oropharynx cancer (71% vs. 29%). In addition, 69% of them had stage IV disease.

**Table 1:** Patient characteristics.

Characteristics	Patients (N = 167), n (%)
Age (years), mean (range)	56 (36–92)
Disease-free survival (years), median (range)	1.92 (0.21–5.53)
Sex	
Male	158 (95)
Female	9 (5)
Performance status (ECOG)	
0	59 (35)
1	102 (61)
2	6 (4)
Primary tumor site	
Oropharynx	48 (29)
Oral cavity	119 (71)
Tumor stage	
I–II	16 (10)
III	35 (21)
IV	116 (69)
Concurrent chemoradiotherapy	120 (72)
Lower-neck irradiation	
Yes	100 (60)
No	67 (40)
Recurrence pattern	
Local Regional lymph node Distant metastasis Total ^a^	34 (20) 26 (16) 13 (8) 55 (33)
Level IV relapse	8 (5)
Radiation dose (cGy) (mean)	
HR-CTV	6,704
IR-CTV	5,997
LR-CTV	5,059

^a^ Some of the patients experienced multiple recurrences, as summarized in Table 3.

ECOG, Eastern Cooperative Oncology Group; HR-CTV, clinical target volume at a high risk; IR-CTV, clinical target volume at an intermediate risk; LR-CTV, clinical target volume at a low risk.

The demographic and baseline clinical characteristics were balanced between the two groups (Table 2). Age, sex, ECOG, tumor stage, recurrence site, and radiation dose were not significantly different between the WNI group and the LNS group. The oropharynx was the primary tumor site for 38% of patients treated with WNI and only 15% of those treated with LNS. In addition, 85% and 52% of the patients in the WNI and LNS groups, respectively, received chemotherapy (*p* < 0.01).

**Table 2:** Comparison of patients treated with WNI and LNS.

Characteristics	Whole-neck irradiation (N = 100)	Lower-neck sparing (N = 67)	*p*-value
Age (years), mean, (range)	55 (39–92)	58 (36–81)	0.1
Sex			0.79
Male	95 (95)	63 (94)	
Female	5 (5)	4 (6)	
Performance status (ECOG)			0.25
0	35 (35)	24 (36)	
1	63 (63)	39 (58)	
2	2 (2)	4 (6)	
Primary site of tumor			<0.01*
Oropharynx	38 (38)	10 (15)	
Oral cavity	62 (62)	57 (85)	
Tumor stage			0.36
I–II	9 (9)	7 (10)	
III	20 (20)	15 (22)	
IV	71 (71)	45 (68)	
Concurrent chemoradiotherapy	85 (85)	35 (52)	<0.01*
Local recurrence			0.19
Yes	17 (17)	17 (25)	
No	83 (83)	50 (75)	
Regional lymph node recurrence			0.12
Yes	12 (12)	14 (21)	
No	88 (88)	53 (79)	
Distant recurrence			0.64
Yes	7 (7)	6 (12)	
No	93 (93)	61 (88)	
Level IV relapse			0.04
Yes	2 (2)	6 (9)	
No	98 (98)	61 (91)	
Radiation dose (cGy), mean			0.53
HR-CTV	6,741	6,604	
IR-CTV	6,014	5,968	
LR-CTV	5,096	4,981	

WNI, whole-neck irradiation; LNS, lower-neck sparing; ECOG, Eastern Cooperative Oncology Group; HR-CTV, clinical target volume at a high risk; IR-CTV, clinical target volume at an intermediate risk; LR-CTV, clinical target volume at a low risk.

Cancer recurrence occurred in 33% of patients, with locoregional recurrence as the most common failure pattern (25%). Distant metastasis with or without locoregional involvement developed in 8% only. Table 3 lists the details of failure patterns. We found 8 (5%) patients who experienced neck level IV recurrence. Among them, 5 had recurrence solely in neck level IV, of which 4 (80%) exhibited solitary recurrence and only 1 (20%) had multiple lymphadenopathies. Furthermore, of those 8 patients who experienced neck level IV recurrence, 2 (25%) received WNI, and 6 (75%) received LNS. We also found that 3 of the 4 patients with solitary recurrence were managed by curative salvage treatment. During the initial diagnosis, only 1 of 8 patients had N0 disease, whereas the rest already had neck nodal metastases. The median time to regional recurrence in level IV was 196 days. Table 4 summarizes the patterns of level IV recurrence in detail.

**Table 3:** Patterns of failure.

Patterns of failure	LNS	WNI
Local and/or regional recurrence	21 (31%)	21 (21%)
Local recurrence only	9 (13%)	11 (11%)
Regional recurrence only	6 (9%)	5 (5%)
Local and regional recurrence	6 (9%)	5 (5%)
Distant metastasis	6 (9%)	7 (7%)
Distant metastasis only	2 (3%)	4 (4%)
Distant metastasis + local recurrence	2 (3%)	1 (1%)
Distant metastasis + regional recurrence	2 (3%)	2 (2%)
Distant metastasis + local recurrence + regional recurrence	0	0

WNI, whole-neck irradiation; LNS, lower-neck sparing.

**Table 4:** Details of level IV recurrence.

No.	Age	Sex	Stage	Primary site	Initial neck level involved	Recurrence site	Time to recurrence (days)	Salvage treatment	Failure pattern
1	56	Male	IVA	Lips	Initial N0	Level IV, solitary	128	Neck dissection	Out-of-field
2	42	Male	IVB	Lips	Level I, II	Level IV pleural seeding	201	Cetuximab	In-field
3	57	Male	IVB	Tongue base	Level I, II, III	Level II, III, IV	475	Cetuximab	In-field
4	67	Male	III	Tongue	Level II, III	Level IV, solitary	574	Neck dissection	Out-of-field
5	78	Male	III	Tongue	Level II	Level IV, solitary	440	Palliation	Out-of-field
6	36	Male	III	Tongue	Level I, II	Level IV, multiple	190	CCRT	Out-of-field
7	37	Male	IVA	Buccal	Level II	Level I, II, III, IV	149	Palliation	Out-of-field
8	81	Male	III	Tonsil	Level II	Level IV, solitary	146	CCRT	Out-of-field

CCRT, concurrent chemoradiotherapy.

**Fig 1:**
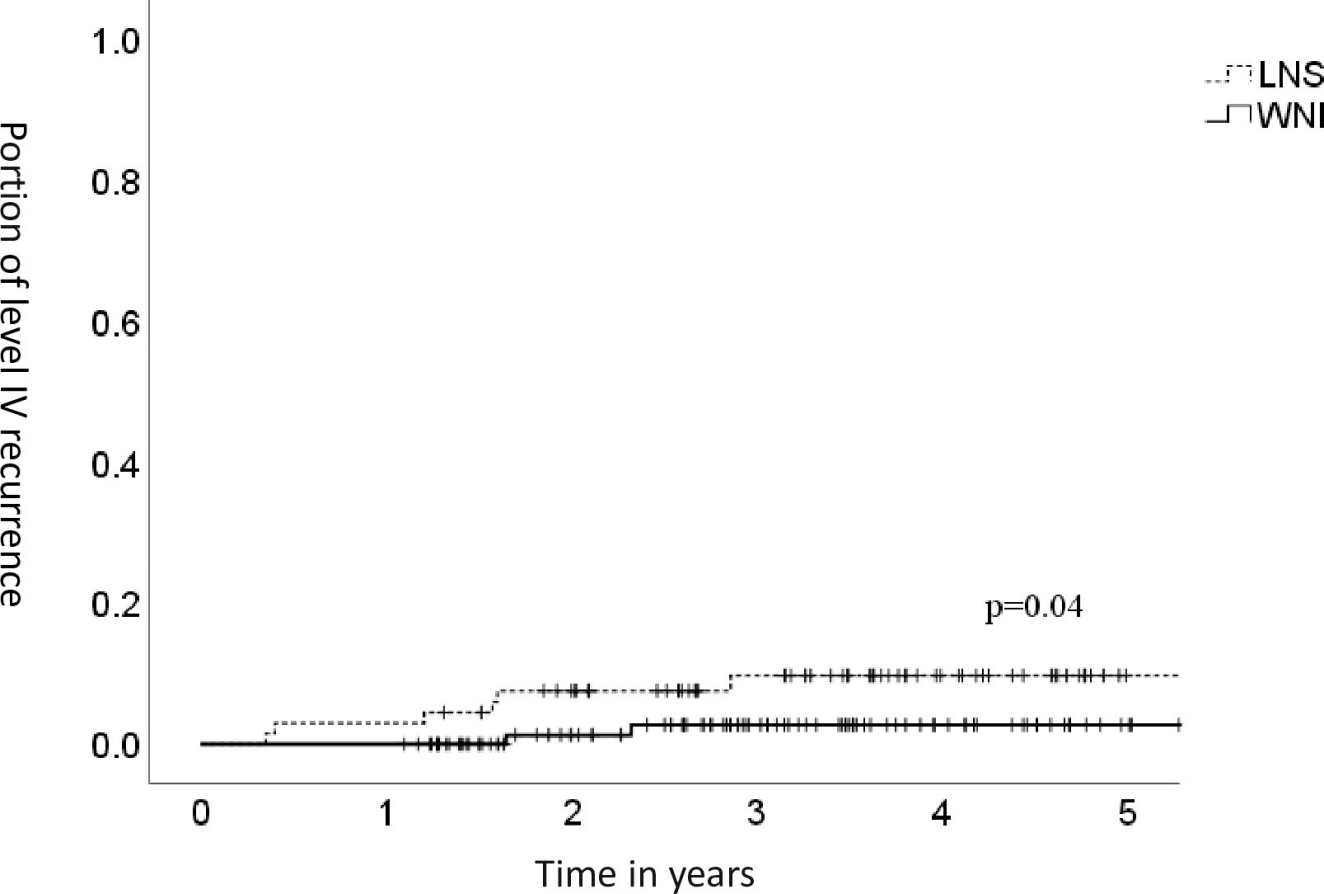
Incidence of level IV recurrence. WNI, whole-neck irradiation; LNS, lower-neck sparing.

**Fig 2:**
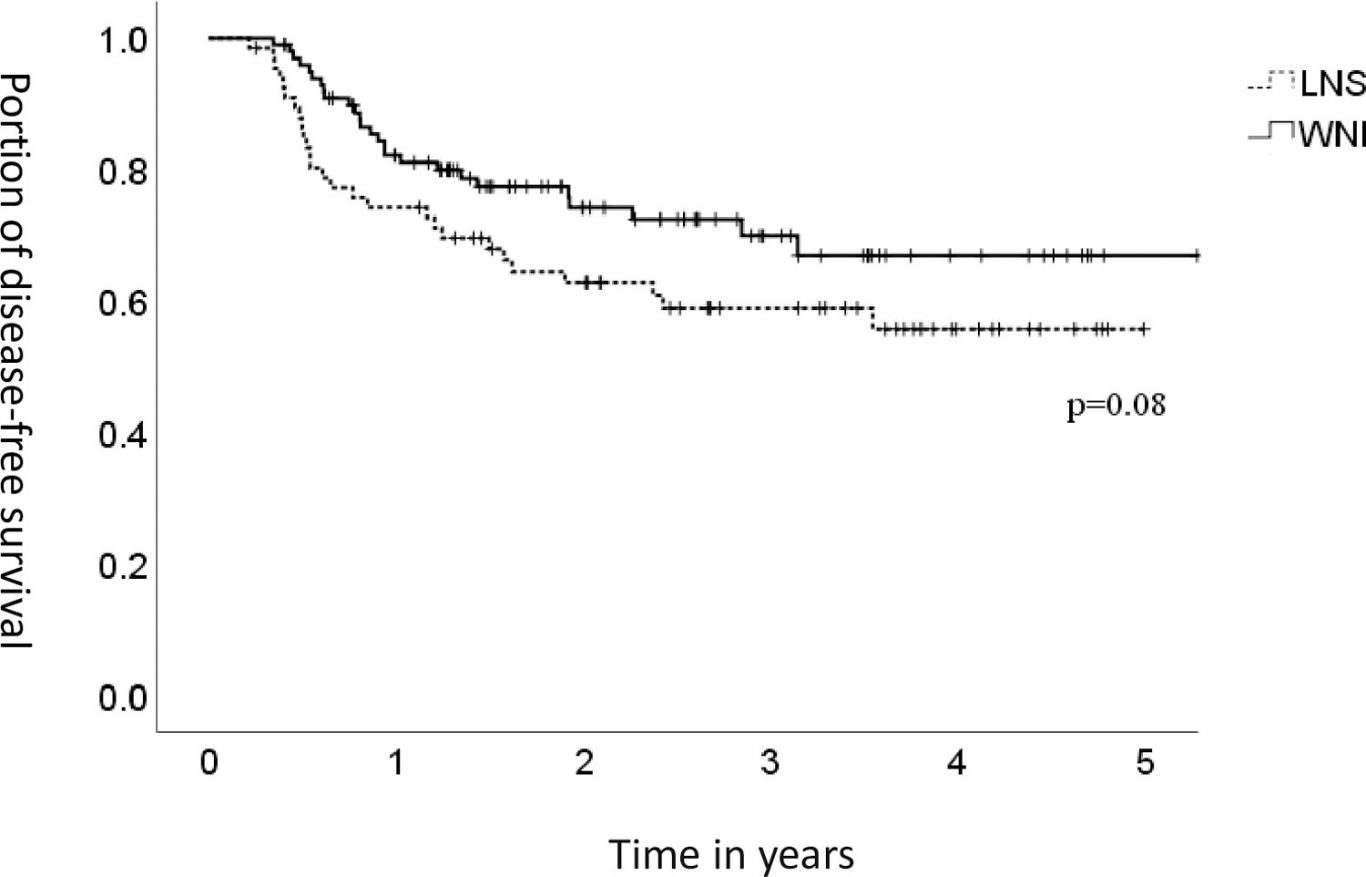
Disease-free survival. WNI, whole-neck irradiation; LNS, lower-neck sparing.

Fig 1 illustrates the incidence of level IV recurrence. The 2-year incidence of level IV recurrence was lower in the WNI group than in the LNS group (2% vs. 10%) (*p* = 0.04). The 2-year DFS was higher in the WNI group than in the LNS group (72% vs. 63%) (*p* = 0.08) (Fig 2). The Cox regression model was applied in the analysis of DFS according to age, sex, performance status, stage, primary tumor site, chemotherapy and lower-neck irradiation (Table 5). Age, sex, chemotherapy, and primary tumor site had no correlation with DFS. Those patients with a poorer performance status (ECOG score of 1–2) obtained an HR of 1.88 on DFS, and those who received lower-neck irradiation had an HR of 0.64 on DFS. In multivariable analysis, the HR was 1.38 in those with a poorer performance status and 0.63 in those who underwent lower-neck irradiation. However, none of them was statistically significant.

**Table 5:** Univariate and multivariate Cox regression analyses of disease-free survival.

	Univariate		Multivariate	
Variable	HR (95% CI)	*p*-value	HR (95% CI)	*p*-value
Age group (years)		0.93		
<55	(Reference)			
≥55	0.93 (0.57–1.68)			
Sex		0.14		
Female	(Reference)			
Male	0.46 (0.17–1.30)			
ECOG		0.04		0.09
0	(Reference)		(Reference)	
1–2	1.88 (1.02–3.47)		1.38 (0.95–2.01)	
Stage		0.19		
I–III	(Reference)			
IV	1.53 (0.82–2.86)			
Primary sites		0.89		
Oral cavity	(Reference)			
Oropharynx	0.98 (0.73–1.32)			
Lower-neck irradiation		0.10		0.09
No	(Reference)		(Reference)	
Yes	0.64 (0.37–1.09)		0.63 (0.37–1.08)	
Chemotherapy		0.90		
No	(Reference)			
yes	1.04 (0.57–1.9)			

HR, hazard ratio; CI, confidence interval; ECOG, Eastern Cooperative Oncology Group.

## Discussion

Either adjuvant radiotherapy or CCRT can effectively prolong DFS and even OS [8,9]. Unfortunately, late toxicity occurs in those with prolonged survival, with more complications caused by radiotherapy. Nevertheless, advances in technique, such as intensity-modulated radiation therapy, volumetric modulated arc therapy, and even proton therapy could dramatically reduce treatment-related toxicity [16–19]. However, lowering the toxicity levels to prevent late toxicity is more important, considering that health professionals value patient’s quality of life. Therefore, a limited irradiation field is being investigated. Many studies focus on omitting lower-neck irradiation for NPC. According to Tang et al., contralateral LNS intensity-modulated radiation therapy seems to be feasible for patients with NPC experiencing unilateral cervical lymph node metastasis because it does not sacrifice nodal relapse-free survival [20]. Sun et al. reported that after the contralateral lower neck was spared, only 4.7% of their patients developed regional recurrence and that all of which occurred within the field only [21]. They concluded that contralateral LNS radiotherapy is safe and feasible, with the potential to improve the long-term quality of life of patients. A meta-analysis conducted by Huang et al. showed that compared with ipsilateral lower-neck prophylactic irradiation, ipsilateral LNS irradiation provided equivalent survival outcomes and regional control in patients with N0–N1 NPC [14].

Compared with NPC, oral cavity cancer treated with LNS irradiation has not yet been reported, and oropharyngeal cancer managed by such method had only one study. In the phase 2 study of David et al., volume de-escalation for elective neck irradiation did not lead to solitary neck recurrence in oropharyngeal and laryngeal cancer [22]. The field of elective neck irradiation included the adjacent cervical lymph node chain if the region involved a node. For example, if level II included a positive node, levels II and III were included in the CTV. Cases of treatment-related dermatitis and gastrostomy use were fewer in that study than in the previous study RTOG 1016. Therefore, the authors concluded that the elective dose and volume reduction was oncologically sound for oropharyngeal and laryngeal cancers, with promising quality-of-life outcomes.

The benefit of LNS has also been discussed by several studies. Sun et al. reported that only 0.4% of their patients developed grade 3 neck fibrosis and 38.6% developed hypothyroidism. Thus, they concluded that omitting the irradiation of the contralateral lower neck decreased the possibility of exposing the dose to the neighboring normal organs and tissues, including the cervical subcutaneous tissues and thyroids [21]. Xiao et al. also noted that patients with omitted lower-neck radiotherapy showed significant decreases in glottic larynx dose and significant improvement in voice quality [15]. Therefore, omitting lower-neck irradiation did not decrease locoregional control but moderately improved treatment-related toxicities in NPC.

A guideline published in 2019 suggests irradiating neck levels I, II, III, and IVa at least in oral cavity cancer and levels II, III, and IVa at least in oropharyngeal cancer [12,23]. However, surgical management largely influences the treatment field of adjuvant radiotherapy. Supraomohyoid selective elective neck dissection is the standard of care for N0 cases, but it could also be considered for N1 and N2 cases [24–26]. Thus, requiring WNI in all patients remains uncertain. Byers et al. evaluated the frequency of skip metastases in 270 patients with tongue tumor [27] and found that only nine patients had metastasis (3.3%) in level IV. Umeda et al. analyzed 247 patients with primary squamous cell carcinoma of the oral mucosa; they discovered that level IV involvement was less than 5% in patients with clinical N0 [28]. Although level IV incidence was low, the 2019 guidelines still recommended irradiation in this area because 10% of patients with pN0 and without postoperative radiotherapy had subsequently developed level IV recurrence [12,27]. In this study, 8% had neck level IV nodal recurrence regardless of whether lower-neck irradiation was provided or not. Half of the patients who developed level IV recurrences showed a solitary disease. Moreover, all those who developed solitary recurrence in level IV were in the LNS group. The result is relatively different from the study reported by David et al. wherein nodal relapse in the omitted area did not occur after volume-reduced irradiation. Although 3 of 4 patients who experienced solitary level IV recurrence were successfully treated by salvage surgery or CCRT, one developed severe pleural seeding, and four had multiple neck nodal recurrence during level IV recurrence, indicating that LNS irradiation is not safe for patients with oral cavity and oropharyngeal cancers.

To our knowledge, this study is the first to discuss the feasibility of LNS irradiation in oral cavity and oropharyngeal cancer. Approximately one third of our patients developed recurrence, with locoregional recurrence and distant failure accounting for 76% and 24% of the recurrences, respectively. The incidence of level IV recurrence was higher in the LNS group. Furthermore, those who underwent WNI exhibited a trend of improvement in 2-year DFS compared with LNS (*p* = .08).

In the univariate and multivariate analyses, age, sex, stage, primary tumor site, performance status, chemotherapy, and lower-neck irradiation did not significantly influence DFS. Although the primary tumor site was not balanced between the two cohorts because of the nature of retrospective data, DFS did not differ between oral cavity and oropharyngeal cancers. RTOG-0129 concluded that HPV-positive oropharyngeal cancer leads to a better outcome than HPV-negative oropharyngeal cancer [29]. According to a nationwide study in Taiwan, the incidence of HPV-positive oropharyngeal cancer is roughly 3% [30,31]. Hence, patients with oropharyngeal cancer in Taiwan did not show a superior outcome. NCCN guideline suggest that standard of care for HPV-positive locally advanced oropharyngeal cancer is definitive concurrent chemoradiotherapy. For, HPV-negative cases, both surgical management and CCRT were recommended. Due to high incidence of HPV-negative disease, surgery plays a more important role in Taiwan.

In the present study, the median follow-up time was below 5 years, which means that it was not long enough to evaluate the overall survival of this cohort. Nonetheless, according to the data from RTOG-9501 and EORTC-22931, the cumulative incidence of local or regional relapses plateaued at approximately 1.5 years during follow-up. Therefore, our median follow-up interval of 3.26 years is still adequate to evaluate the locoregional control.

However, our study has some limitations. First, it is retrospective in design. The details of treatment-related side effect could not be evaluated. Second, the details of pathologic risk factors such as margin status, extranodal extension, lymphovascular invasion, and perineural invasion were unavailable in the cancer registry. Third, the exact nodal metastasis level was not recorded. We could not confirm if lymphadenopathy detected in level II or III shared the same recurrence rate in level IV. Finally, the number of lymph nodes involved by the carcinoma influence recurrence rate in level IV is also uncertain.

## Conclusion

The rate of neck level IV recurrence for oral cavity and oropharyngeal cancers is 5%. The 2-year incidence of level IV recurrence was lower in the WNI group than in the LNS group. Considering the high recurrence rate in oral cavity and oropharyngeal cancers, any modifications in treatment should be performed with care. Trends of improvement in DFS through lower-neck irradiation suggested that it is premature to consider LNS irradiation as daily practice. Further research or even randomized trials are necessary to evaluate the subgroup suitable for LNS irradiation.

## Supporting information

S1 File (XLSX)
